# Supplementary material for: Higher Prevalence of Sarcopenia in Knee Osteoarthritis and Its Association with Femoral Intercondylar Cartilage Thickness and Functional Outcomes
Source: Life (Basel). 2025 Dec 19;16(1):4. doi: 10.3390/life16010004 (PMC12842683; doi:10.3390/life16010004)
Supplement: Supplementary file 1 [file life-16-00004-s001.zip › life-4011569-supplementary.pdf]

Supplementary Table S1. Baseline characteristics of the initial (unmatched) population

Characteristics	Knee OA (n = 136)	Control (n = 138)	p-value
Age (years)	67.31 ± 9.60	65.82 ± 10.42	0.220
Sex, n (%)			
Male	30 (22.1%)	24 (17.4%)	0.413
Female	106 (77.9%)	114 (82.6%)	
Height (cm)	158.26 ± 6.72	159.08 ± 7.10	0.327
Weight (kg)	63.86 ± 10.65	62.03 ± 10.59	0.155
Body mass index (kg/m^2)	21.72 ± 6.27	20.95 ± 4.33	0.237
Dietary habit			
Non-vegetarian	126 (92.6%)	126 (91.3%)	0.852
Vegetarian	10 (7.4%)	12 (8.7%)	
Nutritional supplement use			
Yes	110 (80.9%)	120 (87.0%)	0.499
No	26 (19.1%)	18 (13.0%)	
Comorbidity			
Yes	110 (80.9%)	106 (76.8%)	0.512
No	26 (19.1%)	32 (23.2%)	
Smoking			
Yes	4 (2.9%)	5 (3.6%)	0.989
No	132 (97.1%)	133 (96.4%)	
Alcohol consumption			
Yes	24 (17.6%)	26 (18.8%)	0.921
No	112 (82.4%)	112 (81.2%)	
Regular exercise habit			
Yes	12 (8.8%)	36 (26.1%)	0.001*
No	124 (91.2%)	102 (73.9%)	
